# Prevalent vertebral fracture is dominantly associated with spinal microstructural deterioration rather than bone mineral density in patients with type 2 diabetes mellitus

**DOI:** 10.1371/journal.pone.0222571

**Published:** 2019-09-16

**Authors:** Masahiro Yamamoto, Mika Yamauchi, Toshitsugu Sugimoto

**Affiliations:** Internal Medicine 1, Shimane University Faculty of Medicine, Izumo, Shimane, Japan; Rensselaer Polytechnic Institute, UNITED STATES

## Abstract

**Background:**

An assessment of bone strength based on bone mineral density (BMD) underestimates the risk of fracture in patients with diabetes mellitus (T2DM). However, using the trabecular bone score (TBS) for estimating bone microarchitecture, previous studies showed that bone fragility is associated with deterioration of the microstructure concomitantly with decreased BMD. This study was conducted to clarify which of these skeletal-related factors had a more prominent relationship with bone fragility.

**Research design and methods:**

A retrospective cross-sectional study was performed at Shimane University Hospital. A total of 548 Japanese patients with T2DM [257 postmenopausal women and 291 men aged over 50 years] were included. TBS of the spine was computed from dual-energy X-ray absorptiometry images obtained from BMD measurements.

**Results:**

Vertebral fractures (VFs) were identified in 74 (28.8%) women and 115 (39.5%) men. A relationship between BMD and VFs was observed in the limited subgroup of women with a BMD T-score ≤-1.0. According to multivariate logistic regression analysis, low TBS was significantly correlated with prevalent VFs, independent of BMD in both genders, except for men with a BMD T-score > -1.0. The decision tree showed that the priority factor for determining VFs was TBS, not BMD.

**Conclusion:**

Spinal microarchitecture represented by TBS was a more dominant skeletal factor for bone fragility than the decrease in bone mass, independent of BMD, in patients with T2DM. This observation suggests that loss of structural bone quality was crucial underlying pathogenesis for bone brittleness in these populations, regardless of gender. An integrated assessment of bone strength by BMD and TBS would help diagnose diabetic osteoporosis.

## Introduction

Vertebral fractures (VFs) are a common osteoporotic adverse event and are often asymptomatic [1]. According to two meta-analyses, patients with type 2 diabetes mellitus (T2DM) have a 1.38- and 1.7-fold higher risk of hip fracture than do nondiabetic subjects [2, 3], suggesting that patients with T2DM have an increased risk of fracture. Considering the evidence that VFs are an important sign for the risk of new VFs and nonvertebral fracture in nondiabetic subjects [4, 5], the assessment of bone fragility by prevalent VFs might be useful for the estimation of fracture risk in these patients. However, standard criteria for osteoporosis in patients with T2DM have not been established.

Measurements of bone mineral density (BMD) are the gold standard for evaluating bone strength in patients with primary osteoporosis [6]. However, assessments of bone quantity based on BMD underestimate the risk of fracture in patients with T2DM [2, 7, 8], suggesting that bone fragility in patients with T2DM is caused by poor bone quality [9]. The trabecular bone score (TBS), as computed from conventional BMD images of the lumbar spine captured using dual-energy X-ray absorptiometry (DXA), provides a texture index of spinal trabecular bone that is not captured by standard BMD measurements. Compared with nondiabetic subjects, patients with T2DM of both genders have significantly lower TBS [10–14], except in one study [15]. However, these studies did not provide information on the relationships between TBS and fracture risk. A few reports have identified an association between decreased TBS and an increased risk of fracture, including VFs, in patients with T2DM [15, 16] and populations with diabetes caused by various etiologies, including type 1 DM and steroid treatment [17]. However, these studies were performed in a diabetic population in which fractured patients had a significantly lower BMD than did nonfractured patients [15, 16, 18]. These studies demonstrated that standard BMD measurement can detect bone fragility [15, 17, 18]. These observations raised a question regarding what benefits are provided by measurement of TBS under the condition that BMD can detect bone fragility because these studies have not determined which pathological condition, the loss of bone mass or degeneration of the bone microarchitecture, is a more critical contributor to bone fragility in patients with T2DM.

Recently, the algorithm for evaluating fracture risk in patients with T2DM proposed by the Bone and Diabetes Working Group of the International Osteoporosis Foundation stated that a BMD T-score < -2.0 is the cutoff for starting osteoporosis treatment in patients with diabetes because that value is equivalent to nondiabetes at T-score < −2.5 [19]. In addition, surveillance of bone fragility in these patients is recommended for BMD and TBS. However, this algorithm does not indicate the role of TBS in diagnosing bone fragility in patients with T2DM.

A decision tree analysis is a major data mining method that mathematically builds tree-like classification or regression models by selecting the best relevant factor for predicting the incidence of a target outcome from among various factors. These analyses have been used in the management of public health care and determination of prognostic factors in various diseases [20, 21]. Thus, this evaluation may be useful for revealing which skeletal indices of bone mass or bone microarchitecture primarily affect bone fragility.

To elucidate the impacts of a decreased quantity of bone mass and a deteriorated quality of the bone structure on bone strength, we investigated the association between TBS and the presence of VFs in groups stratified by a BMD T-score = -1.0, which is a cutoff between normal bone and osteopenia, to observe the influence of BMD. In addition, decision tree analysis was performed with factors related to VFs obtained in the present study to determine which bone properties predominantly contributed to increased bone fragility.

## Subjects and methods

### Subjects

Five hundred forty-eight Japanese patients with T2DM [257 postmenopausal women (age range 45–89 years) and 291 men (age range 50–88 years)] who were referred to Shimane University Hospital from community clinics for diabetes treatment and who underwent BMD measurements were consecutively enrolled between 1 April 1998 and 31 December 2013. Their hemoglobin A1c (HbA1c) levels were higher than 6.2% upon enrollment. Patients were excluded if they had higher than normal serum creatinine levels (normal range for women, 0.44–0.83 mg/dL; men, 0.56–1.23 mg/dL); abnormal calcium metabolism, such as primary hyperparathyroidism; a medical checkup history indicating positivity for a glutamic acid decarboxylase antibody; or a history of falls or traffic accidents to eliminate the possibility of injury-associated fractures. The onset of T2DM was defined as the first incident of glucosuria or hyperglycemia. None of the patients were taking any bone-specific drugs or hormones that affected their bone metabolism, including sex steroids, glucocorticoids, warfarin, bisphosphonates, pioglitazone, glucagon-like peptide-1 (GLP-1) agonists, or sodium-glucose cotransporter-2 inhibitors. This study was approved by the Shimane University Institutional Committee on Ethics, a regional ethics board at Shimane University (IRB No. 1427). All procedures performed in studies involving human participants were in accordance with the ethical standards of the institutional and/or national research committee and with the 1964 Helsinki declaration and its later amendments or comparable ethical standards. This study was a retrospective evaluation of routine clinical data. For this type of research, formal consent is not required.

### Biochemical measurements

Serum hemoglobin A1c (HbA1c) was determined by high-performance liquid chromatography. Fasting plasma glucose (FPG), creatinine (Cr), calcium, and phosphate levels were measured in fasting blood samples by using automated techniques at the central laboratory of our hospital as follows: the hexokinase method, enzymatic method, Arsenazo-lll method, and enzymatic method. Serum bone-specific alkaline phosphatase (BAP) and urinary N-telopeptide (uNTX) levels were measured using a commercially available enzyme-linked immunosorbent assay (ELISA).

### Assessment of fractures

In all subjects, lateral X-ray films of the thoracic and lumbar spine were obtained one month before and after the BMD assessment to identify a grade 1 or greater deformity of vertebrae as VFs according to Genant's semiquantitative assessment [22]. These diagnoses were performed by two independent investigators who were blinded to the other's readings. If the investigators did not agree on the presence of VFs, the X-ray film was independently reassessed. If the re-evaluated findings still differed, that case was regarded as no fracture.

### Densitometry

The anteroposterior BMD values of the lumbar spine were measured by DXA using a QDR-4500 system, which was replaced by a Discovery system on 1 October 2008 (Hologic, Waltham, MA, USA). The coefficient of variation for measurements of spinal BMD was 1.0%.

### TBS

Calculations of TBS of the lumbar spine were performed using TBS iNsight software (Version 2.0.0, Med-Imaps, Bordeaux, France), as previously reported (13), with the anteroposterior L1–L4 BMD image (DXA image) obtained from bone densitometry.

### Statistical analysis

All data are presented as the mean ± SD for each index. Mann-Whitney U tests were used to compare the parameters between subjects with and without VFs because these values were not always normally distributed by the Kolmogorov-Smirnov test. Categorical variables were compared using chi-square tests. Logistic regression analyses were performed to determine whether BMD or TBS was associated with VFs independent of the confounding factors that significantly correlated with VFs. Statistical analyses were performed using SPSS software (version 19; IBM Corporation, Tokyo, Japan), except for the decision tree analyses, which were performed using R version 3.4.1 (R Foundation for Statistical Computing) with the classification and regression tree (CART) package. The cutoff values of this analysis were mathematically determined by the CART package based on the Gini coefficient. The Gini coefficient ranges from 0, indicating perfect equality (where each two groups have 50% people with fractures), to 1, perfect inequality (where one group has all people with fracture and the other has people with no fractures). The CART program calculates the Gini coefficient for all variables applied to this package and then chooses the highest Gini coefficient. This Gini coefficient provides the highest performance cutoff value of a certain variable that divides one group into a fracture group and a nonfracture group at the node. *P* values less than 0.05 were considered statistically significant.

## Results

### Characteristics of the study population

The baseline characteristics of the subjects are shown in Table 1. Seventy-four women (28.8%) and 115 men (39.5%) had VFs. HbA1c levels and the duration of T2DM were 9.52 ± 2.09% and 12.6 ± 9.8 years in women and 9.31 ± 2.22% and 12.6 ± 9.7 years in men, respectively. The TBS was 1.236 ± 0.101 in women and 1.322 ± 0.085 in men.

**Table 1:** Background data of men and postmenopausal women with T2DM.

	Women	Men
No. of subjects	257	291
No. of subjects with VF	74 (28.8%)	115 (39.5%)
Age (years)	66.8 ± 9.0	65.3 ± 9.0
BMI (kg/m^2^)	23.6 ± 3.7	23.5 ± 3.5
FPG (mg/dL)	174.7 ± 58.0	172.1 ± 60.7
HbA1c (%)	9.52 ± 2.09	9.31 ± 2.22
Duration of diabetes (years)	12.6 ± 9.8	12.6 ± 9.7
Creatinine (mg/dL)	0.58 ± 0.11	0.76 ± 0.15
Calcium (mg/dL)	9.3 ± 0.4	9.2 ± 0.3
Phosphate (mg/dL)	3.7 ± 0.5	3.3 ± 0.5
BAP (U/L)	33.6 ± 12.5	27.8 ± 10.2
uNTX (nmol BCE/mmol Cr)	56.6 ± 33.0	33.8 ± 17.4
Spine BMD (g/cm^2^)	0.861 ± 0.171	1.054 ± 0.224
T-score	-1.36 ± 1.55	0.06 ± 1.87
Trabecular bone score	1.236 ± 0.101	1.322 ± 0.085
Use of Metformin	71 (27.6%)	58 (19.9%)
Use of sulfonylurea	107 (41.6%)	102 (35.1%)
Use of Insulin	64 (24.9%)	58 (19.9%)

Data are expressed as the mean ± SD. T2DM, type 2 diabetes; VF, vertebral fracture; BMI, body mass index; FPG, fasting plasma glucose; BAP, bone-specific alkaline phosphatase; uNTX, urinary levels of N-telopeptide; BCE, bone collagen equivalents.

### Comparison of various parameters between patients with and without VFs among all participants

Compared with patients without VFs, patients of both genders with VFs were significantly older (*P* < 0.01) and had a significantly lower TBS (*P* < 0.01) (Tables 2 and 3). Only women with VFs presented significantly lower spinal BMD and T-scores than did women without VFs (*P* < 0.05). Significantly lower serum phosphate levels (*P* < 0.05) and a significantly longer duration of T2DM (*P* < 0.05) were observed in women with VFs than in women without VFs.

**Table 2:** Comparison of various parameters between patients with T2DM presenting with and without vertebral fractures in women.

	All subjects		BMD T-score ≤ -1.0		BMD T-score > -1.0	
	Vertebral fractures		Vertebral fractures		Vertebral fractures	
	No	Yes	No	Yes	No	Yes
Factors	Mean (SD)	Mean (SD)	Mean (SD)	Mean (SD)	Mean (SD)	Mean (SD)
No. of subjects	183	74	107	51	76	23
Age (years)	65.6 (88.9)	69.7 (8.6) **	67.6 (8.4)	70.9 (8.2) *	62.7 (8.9)	67.0 (9.1) *
BMI (kg/m^2^)	23.7 (3.6)	23.4 (3.9)	23.1 (3.3)	22.9 (3.9)	24.5 (3.8)	24.3 (3.8)
FPG (mg/dL)	171.8 (58.9)	182.0 (55.6)	173.3 (60.0)	180.6 (55.6)	169.6 (57.7)	185.4 (56.7)
HbA1c (%)	9.44 (2.04)	9.70 (2.23)	9.20 (2.10)	9.64 (2.24)	9.78 (1.92)	9.84 (2.24)
Duration of diabetes (years)	11.6 (9.3)	14.9 (10.6) *	12.8 (9.6)	14.4 (10.3)	10.0 (8.7)	15.8 (11.3) *
Creatinine (mg/dL)	0.58 (0.11)	0.56 (0.10)	0.59 (0.11)	0.56 (0.10)	0.57 (0.11)	0.58 (0.09)
Calcium (mg/dL)	9.3 (0.4)	9.3 (0.3)	9.3 (0.4)	9.3 (0.3)	9.4 (0.4)	9.3 (0.4)
Phosphate (mg/dL)	3.8 (0.5)	3.6 (0.5) *	3.7 (0.5)	3.5 (0.6)	3.8 (0.5)	3.7 (0.5)
BAP (U/L)	32.7 (11.7)	35.8 (14.1)	35.0 (12.7)	37.7 (14.2)	29.1 (9.0)	31.1 (13.1)
uNTX (nmol BCE/ mmol Cr)	54.4 (28.0)	61.6 (42.0)	58.2 (30.5)	67.0 (47.1)	48.4 (22.4)	48.9 (22.4)
Spine BMD (g/cm^2^)	0.876 (0.161)	0.824 (0.190) *	0.769 (0.088)	0.729 (0.120) *	1.028 (0.111)	1.035 (0.140)
T-score	-1.23 (1.46)	-1.69 (1.72) *	-2.21 (0.79)	-2.55 (1.09) *	0.15 (1.00)	0.22 (1.26)
Trabecular bone score	1.252 (0.103)	1.195 (0.083) **	1.211 (0.091)	1.172 (0.080) *	1.310 (0.091)	1.246 (0.068) **
Use of Metformin	53 [29.0%]	18 [24.3%]	30 [28.0%]	10 [19.6%]	23 [30.3%]	8 [34.8%]
Use of sulfonylurea	78 [42.6%]	29 [39.2%]	43 [40.2%]	24 [47.4%]	35 [46.1%]	5 [21.7%]
Use of Insulin	42 [23.0%]	22 [29.7%]	27 [25.2%]	13 [26.4%]	15 [19.7%]	9 [39.1%]

Mann-Whitney U tests:

* *P* < 0.05 and

** *P* < 0.01

compared with each non-VF group

T2DM, type 2 diabetes; VF, vertebral fracture; BMI, body mass index; FPG, fasting plasma glucose; BAP, bone-specific alkaline phosphatase; uNTX, urinary levels of N-telopeptide; BCE, bone collagen equivalents.

**Table 3:** Comparison of various parameters between patients with T2DM presenting with and without vertebral fractures in men.

	All subjects		BMD T-score ≤ -1.0		BMD T-score > -1.0	
	Vertebral fractures		Vertebral fractures		Vertebral fractures	
	No	Yes	No	Yes	No	Yes
Factors	Mean (SD)	Mean (SD)	Mean (SD)	Mean (SD)	Mean (SD)	Mean (SD)
No. of subjects	176	115	52	34	124	81
Age (years)	63.7 (8.7)	67.9 (8.8) **	63.5 (9.2)	67.3 (9.9)	63.8 (8.5)	68.1 (8.3) **
BMI (kg/m^2^)	23.5 (3.6)	23.5 (3.3)	23.1 (3.3)	22.9 (3.9)	24.0 (3.7)	23.9 (3.1)
FPG (mg/dL)	176.6 (61.9)	164.8 (58.2)	185.2 (66.4)	168.6 (61.0)	173.2 (59.9)	163.5 (57.6)
HbA1c (%)	9.50 (2.38)	9.00 (1.90)	9.33 (2.40)	9.11 (1.93)	9.57 (2.38)	8.97 (1.91)
Duration of diabetes (years)	12.7 (9.8)	12.5 (9.5)	10.5 (9.7)	13.4 (10.8)	13.6 (9.7)	12.2 (8.9)
Creatinine (mg/dL)	0.77 (0.16)	0.76 (0.14)	0.74 (0.17)	0.74 (0.15)	0.78 (0.15)	0.77 (0.14)
Calcium (mg/dL)	9.2 (0.3)	9.2 (0.4)	9.2 (0.3)	9.2 (0.3)	9.3 (0.4)	9.2 (0.4)
Phosphate (mg/dL)	3.4 (0.4)	3.3 (0.5)	3.4 (0.4)	3.5 (0.5)	3.3 (0.4)	3.2 (0.5) *
BAP (U/L)	27.7 (10.6)	27.9 (9.7)	27.3 (11.1)	29.0 (11.8)	27.8 (10.4)	27.4 (8.6)
uNTX (nmol BCE/ mmol Cr)	33.5 (17.3)	34.1 (17.7)	36.9 (16.2)	39.1 (18.0)	32.0 (17.6)	32.1 (17.3)
Spine BMD (g/cm^2^)	1.053 (0.215)	1.055 (0.237)	0.830 (0.072)	0.805 (0.095)	1.146 (0.184)	1.162 (0.19)
T-score	0.04 (1.80)	0.08 (1.97)	-1.82 (0.61)	-2.04 (0.80)	0.82 (1.53)	0.99 (1.61)
Trabecular bone score	1.334 (0.080)	1.304 (0.089) **	1.299 (0.070)	1.258 (0.087) *	1.349 (0.079)	1.323 (0.084) *
Use of Metformin	38 [21.6%]	20 [17.4%]	3 [5.9%]	5 [14.7%]	35 [28.2%]	15 [18.5%]
Use of sulfonylurea	62 [35.2%]	40 [34.8%]	16 [30.8]	10 [29.4%]	46 [37.0%]	30 [37.0%]
Use of Insulin	35 [19.9%]	23 [20.0%]	10 [16.2%]	8 [23.5%]	25 [20.2%]	15 [28.5%]

Mann-Whitney U tests:

* *P* < 0.05 and

** *P* < 0.01, compared with each non-VF group

T2DM, type 2 diabetes; VF, vertebral fracture; BMI, body mass index; FPG, fasting plasma glucose; BAP, bone-specific alkaline phosphatase; uNTX, urinary levels of N-telopeptide; BCE, bone collagen equivalents.

### Comparison of various parameters between patients with and without VFs in the subgroup stratified by a spinal BMD T-score of -1.0

Participants were divided into subgroups based on a BMD T-score of -1.0 to exclude the effect of the BMD on the association between the TBS and the presence of VFs (Tables 2 and 3). A significantly lower TBS was recorded for patients of both genders in the VF group than for patients in the non-VF group regardless of the T-score subgroups. In contrast, a significantly lower BMD was observed only in women with VFs in BMD T-score ≤ -1.0. Patients in the VF group were significantly older than those in the non-VF group, except among men with a T-score ≤ -1.0. In the subgroup with a BMD T-score > -1.0, the duration of T2DM in women with fracture was significantly higher than that in those without fracture. The serum phosphate level in men with fracture was significantly lower than that in those without fracture.

### Association between the presence of VFs and BMD or TBS

**Fig 1:**
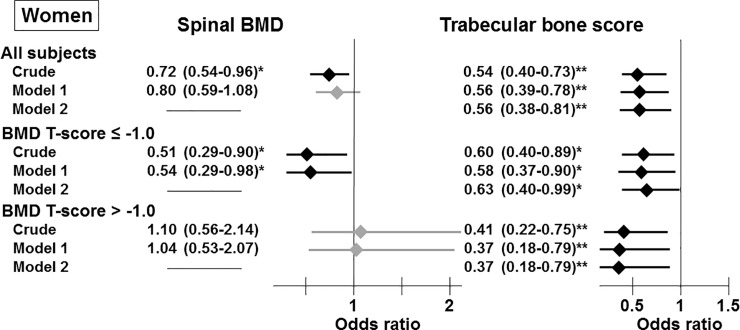
Associations between the presence of vertebral fractures and spinal BMD and TBS in all participants and the subgroups stratified by spinal BMD T-scores in women. Odds ratios are presented as a 1 SD increase in spinal BMD and TBS. Model 1: adjusted for age, the duration of T2DM, and phosphate levels. Model 2: adjusted for the factors included in Model 1 and spinal BMD. *, *P* < 0.05; **, *P* < 0.01.

**Fig 2:**
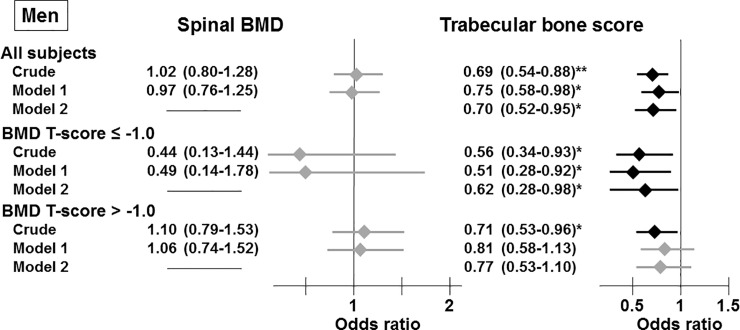
Associations between the presence of vertebral fractures and spinal BMD and TBS in all participants and the subgroups stratified by spinal BMD T-scores in men. Odds ratios are presented as a 1 SD increase in spinal BMD and TBS. Model 1: adjusted for age, the duration of T2DM, and phosphate levels. Model 2: adjusted for the factors included in Model 1 and spinal BMD. *, *P* < 0.05; **, *P* < 0.01.

To clarify the association between bone fragility and bone-related indices, such as the BMD or TBS, we performed logistic regression analyses to investigate the relationships between the presence of VFs, an indicator of bone fragility, and BMD as well as TBS. In women, BMD was significantly correlated with the presence of VFs in the crude model in the group composed of all subjects and the subgroup with a BMD T-score ≤ -1.0 (Fig 1). However, this significant association disappeared after adjustment for the confounding factors for VFs revealed by Tables 1 and 2, such as age, the duration of T2DM, and phosphate levels (Model 1), in all subjects. Nevertheless, this correlation remained significant in women with a BMD T-score ≤ -1.0 after adjusting for the factors included in Model 1. However, a significant relationship between the BMD and the presence of VFs was not observed in any men (Fig 2). In addition, these findings were unaffected by an additional adjustment for the hypoglycemic agent (data not shown).

In contrast to BMD, a low TBS was significantly and consistently correlated with an increased risk of the presence of VFs in the crude estimation and Model 1 in both genders in any group (Figs 1 and 2), except for men with a BMD T-score > -1.0 (Fig 2). In addition, these significant relationships were observed after additional adjustment for BMD (Model 2).

### Decision tree analysis for the determination of the prevalence of VFs in patients with T2DM

**Fig 3:**
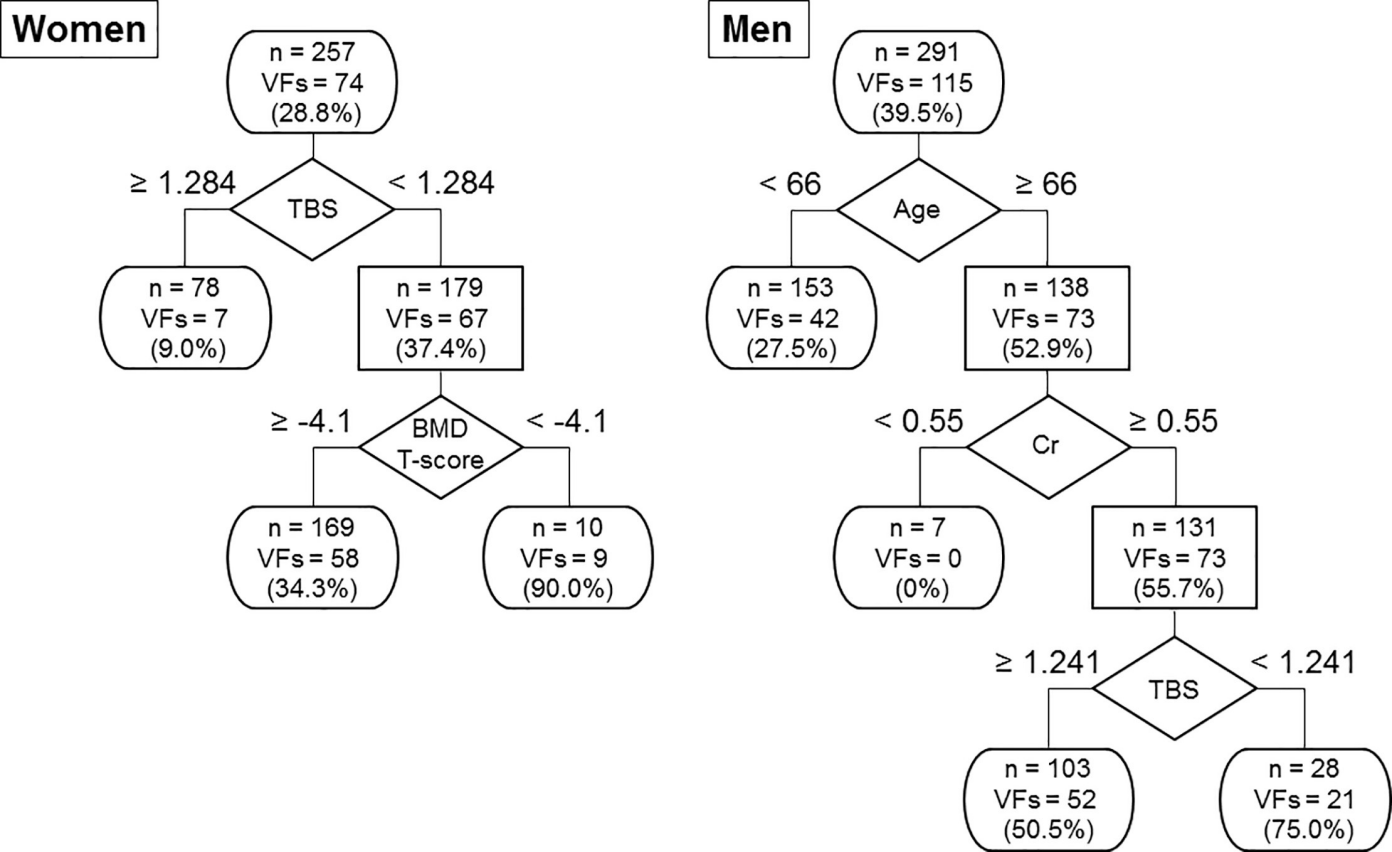
Decision tree analysis for the determination of the prevalence of vertebral fractures in patients with T2DM.

The decision trees were constructed to determine the crucial factors for the incidence of VFs in descending order with all variables listed in Table 1, including the confounding factors shown in Fig 1: age, duration of T2DM, phosphate level, TBS, and T-score instead of BMD (Fig 3). At each level of bifurcation point in the tree-like chart, the best splitter to determine the presence of VFs was placed by a mathematical technique. The number of subjects and patients with VFs were described in each node or terminal. TBS was the top priority factor that differentiated female patients with VFs from those without VFs and was superior to BMD. In male patients, TBS was selected as the third factor for determination of the presence of VFs following age and creatinine levels.

The objective groups were classified into two subgroups based on the cutoff values of the given factors that minimized the variation in the fracture rate. The number of subjects and patients with VFs was described in each node or terminal.

## Discussion

For the first time, the present study demonstrated that TBS was more definitive than BMD in discriminating patients with the presence of VFs by decision tree analysis. This observation was validated by the findings that the consistent and significant relationships between lower TBS and the presence of VFs were independent of BMD in both genders, except for men with a T-score > -1.0.

This study showed that TBS was chosen prior to BMD as an upper node of the decision trees for the prevalence of VFs in both genders. Decision trees, which are an essential component to establishing artificial intelligence (AI), such as the random forest approach, have been widely applied to computational biology and bioinformatics because of their usefulness in aggregating diverse types of data to make accurate predictions. Decision trees inform us which variables more profoundly contribute to the outcomes than others because tree-shaped charts were built by preferentially selecting the most relevant variables that are associated with target outcome. Currently, which bone component, bone mass and microarchitectures, has more critical impacts on bone fragility in patients with T2DM is unclear because previous studies did not validate the confounding effect of BMD on fracture risk [15, 16, 18]. However, for the first time, the present observations obtained by decision tree analyses indicated that lower TBS was a more powerful determining factor of bone fragility than the decrease in BMD in patients with T2DM.

Compared with decision tree analyses, logistic regression analyses were useful to determine the causes of bone fragility in this population. The assessment of bone fragility using the full range of BMD did not reflect the bone strength of the patients with T2DM, similar to previous studies [2, 7, 8]. In addition, bone strength in patients with T2DM is estimated at equivalent to bone fragility whose T-score is approximately 0.5 units lower than that in women without diabetes [7]. In contrast, TBS was significantly and consistently associated with the presence of VFs in women. In addition, a similar finding was obtained in men with a BMD T-score ≤ -1.0 for the first time. These relationships were independent of BMD as shown in a previous report [17], suggesting that TBS might be an index reflecting structural bone quality, such as deterioration of the trabecular microarchitecture. Additionally, one recent study demonstrated that TBS possesses a nature sensitive to multiple risk factors for fracture, including the presence of diabetes [23]. Thus, TBS, not BMD, was robustly associated with the presence of VFs in patients with T2DM, supporting the results obtained from the decision trees in this study. These findings indicated that deterioration of bone microarchitecture, one component of bone quality, was a more dominant skeletal cause of bone fragility than the decrease in bone mass in patients with T2DM.

Here, the relationship between the presence of VFs and lower BMD was observed in limited subgroups, such as women with a BMD T-score ≤ -1.0, a threshold value for osteopenia, even though bone fragility is not easily determined by BMD in the typical population of patients with T2DM. As the BMD T-score increased, the trends of the hip fracture risk in patients with T2DM decreased [7], indicating that the risks of fracture in patients with T2DM are affected by BMD. However, those decreased fracture risks equal the risk of the population without T2DM [7], suggesting that the elevated relative risk of fracture in patients with T2DM compared to that in controls cannot always be explained by BMD, especially in the patients with T2DM whose BMD is within the normal range. In addition, meta-analysis showed that individuals with T2DM have higher BMD levels than do controls [24], suggesting that BMD in a typical T2DM population might be distributed in a higher range, which is not commonly considered to be the cause of fracture. Taking into consideration the results obtained from the present study, logistic regression analyses confirmed that evaluation of fracture risk by BMD was slightly useful in patients with T2DM, supporting the result demonstrated by the decision tree analyses that BMD was not the best factor for determining the presence of VFs.

This study has some limitations. First, this study was not conducted with a sufficient number of population-based subjects to draw definitive conclusions. Second, the patients enrolled in this study might have had relatively severe cases of T2DM and might not be representative of typical Japanese patients with T2DM, as the diabetic conditions of the patients who attended Shimane University Hospital, a tertiary care center, were considered to be more severe than those of other patients with T2DM. Third, T2DM is a complex disease concomitant with various metabolic disorders, and some of these disorders affect bone metabolism. This study could not provide background data affecting osteoporosis, such as serum levels of vitamin D and parathyroid hormone (PTH) and diabetes-related risk factors for fractures, such as advanced glycation end-products (AGEs) [25, 26], endogenous secretory receptors for AGEs (esRAGE) [27], and sclerostin [28, 29], on the relationship between TBS and VFs. Fourth, the patients with osteoarthrosis and lumbar scoliosis, which confound DXA measurements, were not excluded. A study revealed that the severity of spine osteoarthrosis did not correlate with TBS in contrast to BMD [30]. Thus, the study protocol with easy clinical use, as in this study, was potentially biased toward the diminishing ability to determine bone strength by BMD. Fifth, this study did not consider the influence of the heterogeneous distribution of BMD or TBS inside the vertebral body on bone strength. A recent study, which was not performed with subjects with diabetes, indicated that subregional areal BMD reflects variations in "real" trabecular bone microstructure that were better determined by high-resolution peripheral quantitative computed tomography (CT) than by subregional TBS [31]. Thus, BMD in certain local areas has the potential to be a helpful indicator of bone fragility in diabetic conditions. Sixth, information on the cross calibration of TBS is not available. Finally, this study did not investigate the effects of other T-scores, such as -2.5 or -2.0, which are the cutoff values for osteoporosis determined by BMD [32] and the proposed value for starting osteoporosis treatment in patients with T2DM [19], on the relationship between TBS and the presence of VFs because the number of subgroups was too small to perform logistic regression analyses. For the same reason, the limited sample size might have an effect on the results of women with a T-score >-1.0 obtained from the logistic regression analysis because of the small sample size (n = 23).

In conclusion, the decision tree analyses revealed that spinal microarchitecture represented by TBS was a more dominant skeletal factor for bone fragility than the decrease in bone mass, independent of BMD, in patients with T2DM. In contrast, BMD measurement might be useful in women or in the limited BMD range, such as osteopenia or osteoporosis. These observations suggest that loss of structural bone quality was a crucial underlying pathogenesis for bone brittleness in these populations, regardless of gender. An integrated assessment of bone strength by BMD and TBS would help diagnose diabetic osteoporosis.
